# Recruitment of Perisomatic Inhibition during Spontaneous Hippocampal Activity *In Vitro*

**DOI:** 10.1371/journal.pone.0066509

**Published:** 2013-06-21

**Authors:** Anna Beyeler, Aude Retailleau, Colin Molter, Amine Mehidi, Janos Szabadics, Xavier Leinekugel

**Affiliations:** 1 Université de Bordeaux, Institut des Maladies Neurodégénératives, UMR 5293, Bordeaux, France; 2 Centre National de la Recherche Scientifique, Institut des Maladies Neurodégénératives, UMR 5293, Bordeaux, France; 3 European Network Institute, Bordeaux, France; 4 Institute of Experimental Medicine, Hungarian Academy of Sciences, Budapest, Hungary; University of Bristol, United Kingdom

## Abstract

It was recently shown that perisomatic GABAergic inhibitory postsynaptic potentials (IPSPs) originating from basket and chandelier cells can be recorded as population IPSPs from the hippocampal pyramidal layer using extracellular electrodes (eIPSPs). Taking advantage of this approach, we have investigated the recruitment of perisomatic inhibition during spontaneous hippocampal activity *in vitro*. Combining intracellular and extracellular recordings from pyramidal cells and interneurons, we confirm that inhibitory signals generated by basket cells can be recorded extracellularly, but our results suggest that, during spontaneous activity, eIPSPs are mostly confined to the CA3 rather than CA1 region. CA3 eIPSPs produced the powerful time-locked inhibition of multi-unit activity expected from perisomatic inhibition. Analysis of the temporal dynamics of spike discharges relative to eIPSPs suggests significant but moderate recruitment of excitatory and inhibitory neurons within the CA3 network on a 10 ms time scale, within which neurons recruit each other through recurrent collaterals and trigger powerful feedback inhibition. Such quantified parameters of neuronal interactions in the hippocampal network may serve as a basis for future characterisation of pathological conditions potentially affecting the interactions between excitation and inhibition in this circuit.

## Introduction

Feedback inhibition is the inhibition triggered by the network over itself, following activation of excitatory neurons. It is considered to be of primary importance in the CA3 hippocampal circuit to support network oscillations[1]–[8] as well as to avoid hyper-synchronisation (which ultimate form is seizures) due to recurrent excitation by pyramidal cells local collaterals[9]–[14]. Among the various types of GABAergic interneurons present in the hippocampus [3], [5], [15], previous studies have suggested that perisomatic basket and chandelier cells provide strong and rapid monosynaptic inhibition to pyramidal cells[5], [10], [16]–[20]. However, basic parameters regarding the integration of excitation and perisomatic inhibition in this circuit have not been described during spontaneous activity, although they play a predominant role in shaping neuronal output of the recurrent CA3 circuit and are critical parameters for the development of realistic hippocampal models. Taking advantage of an original approach which consists in the extracellular detection of perisomatic inhibitory events from their target population (extracellular inhibitory post-synaptic potentials, eIPSPs [21], [22]), we could address experimentally the dynamics of the interactions between excitation and inhibition in the CA3 circuit. How precisely in time does inhibition control pyramidal cells firing? Previous work in vitro suggested that CA3 pyramidal cells hardly recruit each other in the presence of functional inhibition, most pyramidal spikes being fired without preceding excitatory post-synaptic potentials [17]. Given the low connectivity between excitatory cells and the high level of inhibition in the hippocampal network [5], [11], to what extent does neuronal recruitment through local recurrent excitatory collaterals operate during spontaneous activity, in the presence of functional inhibition?

## Results

### Extracellular Recording of Perisomatic IPSPs in CA3

To investigate how excitatory activity is integrated into feedback inhibitory networks, and the precise effect of interneuronal discharge on the timing of pyramidal cells’ spikes during spontaneous activity in the CA3 hippocampal circuit, we performed extracellular recordings of perisomatic inhibitory events from their target population (extracellular inhibitory post-synaptic potentials, eIPSPs), as described in recent publications [21], [22].

**Figure 1:**
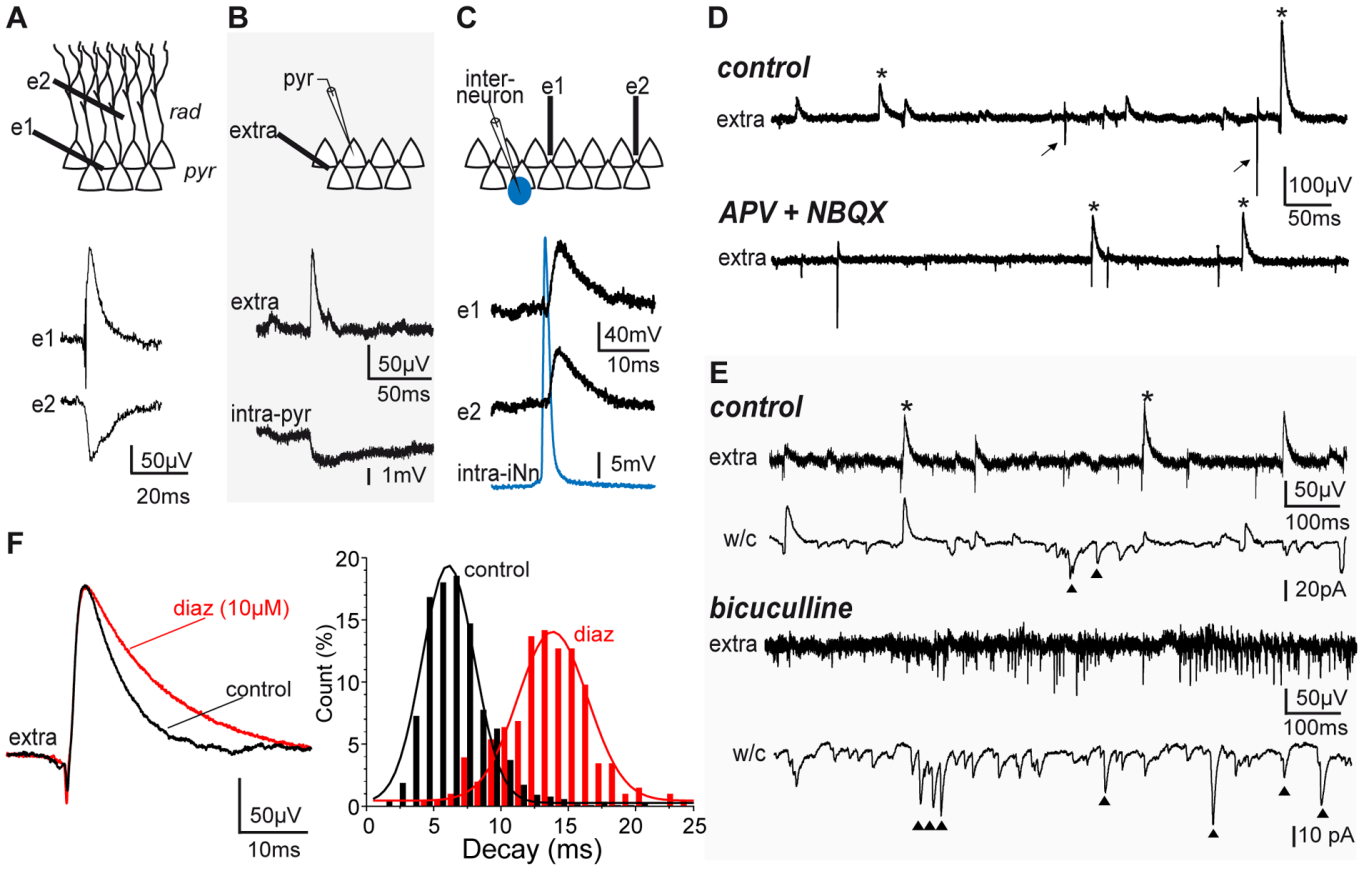
Extracellular recording of spontaneous inhibitory post-synaptic potentials (eIPSPs) in CA3. A. Simultaneous extracellular recording (guinea pig) from CA3 strata pyramidale (**e1**, **pyr**) and radiatum (**e2**, **rad**) of spontaneous eIPSPs in vitro. Note eIPSP reversed polarity in radiatum. B. Simultaneous intracellular (from a pyramidal neuron, **intra-pyr**) and extracellular (**extra**) recording of an inhibitory synaptic event (guinea pig). C. Simultaneous extracellular recording (**e1** and **e2**, separated by about 150 µm) of eIPSPs evoked by the spontaneous firing of a single action potential from an intracellularly recorded interneuron (**intra-iNn**), which soma was located at the oriens/pyramidale border (guinea pig). D. Spontaneous extracellular activity in the pyramidal layer (guinea pig): eIPSPs, of positive polarity (upper trace, *), are easily distinguishable from multi-unit activity (**arrows**). Upper trace, control. Lower trace, in presence of the NMDA and AMPA/KA glutamatergic receptor blockers APV+NBQX. E. Spontaneous activity (**control**) simultaneously recorded in extracellular (**extra**) and patch clamp (**w/c**, whole cell voltage clamp at −50 mV from an individual nearby pyramidal neuron) in a P21 rat. Due to ECl-, GABA-A currents are outward while glutamatergic currents (**triangles**) are inward. Note that in presence of bicuculine (bicu), eIPSPs and IPSPs are no longer observed, in spite of intense neuronal activity visible as multi-unit discharge (upper trace, **extra**) and high-frequency glutamatergic synaptic events (lower trace, **w/c**, **triangles**). F. Comparison of eIPSP decay (left, average trace normalized on amplitude, right, histogram distribution of eIPSPs decay times) in control (black) and in presence of diazepam (red) in a guinea pig. Note prolonged eIPSP decay under diazepam.

**Figure 2:**
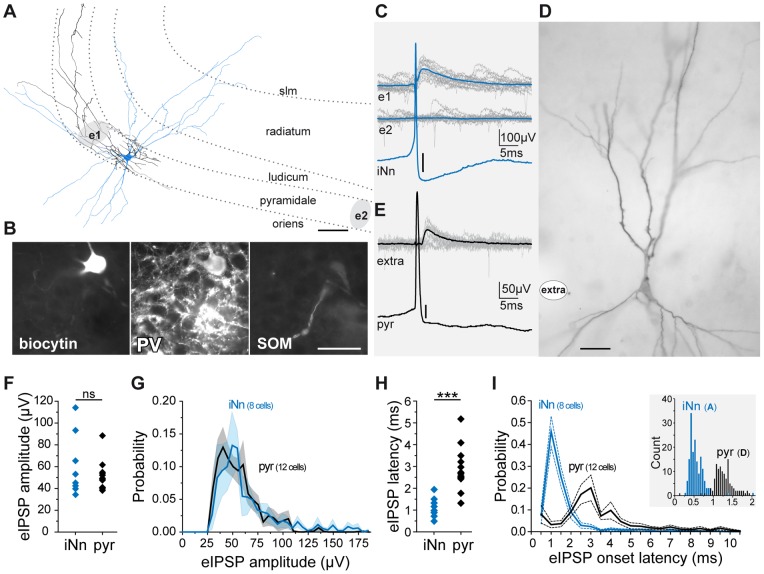
eIPSPs induced by single interneurons and pyramidal cells in CA3. A–E. eIPSPs triggered by the discharge of single action potentials by a CA3 parvalbumin positive basket cell and by a pyramidal cell. Both neurons were recorded intracellularly in the CA3 pyramidal layer of rat slices and filled with biocytin. A. Camera lucida drawing of the axon (black) and dendrites (blue) of the interneuron and position of the extracellular electrodes (grey areas e1 and e2); scale bar 25 µm; dotted lines indicate the borders of the hippocampal strata: lacumosum-moleculare (slm), radiatum, lucidum, pyramidale and oriens. B. Immunohistochemical characterization of the same cell; scale bar 50 µm. Note perisomatic projection (A), parvalbumin positive (B, PV) and somatostatin negative (B, SOM) labelling. C. A single action potential from this cell (c bottom, averaged trace, scale bar 3 mV) was associated with an eIPSP in the CA3 pyramidal layer at the recording site corresponding to the interneuron projection area (e1, upper traces, 15 superimposed sweeps) but not at another recording site outside this area (e2, lower traces). D. photograph of the recorded and biocytin labeled pyramidal cell; scale bar 20 µm; **white spot**, extracellular recording site. Note the thorny excrescences on the proximal apical dendrites. E. eIPSPs (upper traces, 15 superimposed sweeps) triggered by the discharge of single action potentials (bottom, averaged trace, scale bar 10 mV) by the morphologically identified CA3 pyramidal cell shown in D. F–I. Amplitudes and latencies of eIPSPs evoked by the discharge of single spikes from individual interneurons (iNn) and pyramidal cells (pyr) recorded intracellularly. F. Average amplitude (one point per presynaptic neuron) of the evoked eIPSPs. G. Distribution of the amplitudes (mean±SEM) of the evoked eIPSPs. H. Average latencies (one point per presynaptic neuron) of the evoked eIPSPs. I. Distribution of the latencies (mean±SEM) of the evoked eIPSPs. Note that the distribution of interneuronal spikes to eIPSP latencies suggests monosynaptic transmission, while the longer latencies of eIPSPs evoked by pyramidal cells spikes suggest disynaptic transmission. Inset shows example histograms of eIPSP latencies for the interneuron shown in A (iNnd, blue bars) and for the pyramidal cell shown in D (pyr, black bars).

When tungsten microelectrodes were inserted in the CA3 pyramidal layer of hippocampal slices, spontaneous local field potentials (LFP) of positive polarity were recorded (Fig. 1), in addition to multi-unit activity. In keeping with recently published data, these events likely correspond to the GABA-A receptor (GABA-A R) mediated synaptic currents evoked in their pyramidal target population by the synchronous release of GABA from the multiple synaptic terminals of perisomatically projecting interneurons. First, they often (41±20%, n = 12 cells from 8 animals) correlated with inhibitory postsynaptic signals from an individual pyramidal cell recorded intracellularly (Fig. 1B), their kinetics (rise time 2.06±0.47 ms, mono-exponential decay time constant 5.23±2.56 ms, n = 3578 and 3292 events respectively, from 6 animals, Fig. 1F) were similar to those of GABA-A mediated IPSCs, and they showed reversed polarity in the stratum radiatum (Fig. 1A) as reported for basket and chandelier cells mediated events [21], [22]. Second, they persisted in the presence of the glutamatergic NMDA and AMPA/KA receptor antagonists, APV (100 µM) and NBQX (10 µM) or DNQX (5 µM), which did not significantly change their amplitudes (49.8±14.9 µV in control, 44.3±10.2 µV in APV+NBQX/CNQX, n = 5 recordings from 3 rats, p>0.05, paired Student’s T test) although their frequency decreased by 75% from 3.77±1.4 to 1.39±1.6 events/s (n = 5 recordings from 3 rats, p<0.05, paired Student’s T test, Fig. 1D). Third, they were not observed in presence of the selective GABA-A R antagonist bicuculline (20 µM, n = 3). Indeed, glutamatergic activity alone (indicated by both intense extracellular multi-unit activity and patch-clamp whole-cell recordings in the presence of bicuculline) did not produce such LFPs (Fig. 1E). Fourth, application of diazepam (10 µM), which selectively prolongs the decay of GABA-A R mediated synaptic currents [24], produced a 70% increase of average decay time constants for eIPSPs (from 5.81±2.54 to 9.87±4.28 ms, p<0.05, n = 5045 and 2726 events respectively, from 4 slices), indicating that they are indeed mediated by GABA-A R (Fig. 1F). And finally, as also reported previously [21], [22], recording intracellularly from interneurons whose somata were located in or adjacent to the pyramidal layer, we occasionally (n = 11 cells) found neurons in which firing of a single action potential was sufficient to evoke an eIPSP in the extracellular record over distances of several tens of µm (Fig. 1C). Among five eIPSP triggering interneurons filled with either biocytin or neurobiotin, three could be successfully processed for morphological and immunocytochemical analysis. All showed typical basket cell morphology, with perisomatic axonal projection and positive immuno-staining for parvalbumin but not for somatostatin (Fig. 2A,B). As illustrated in Fig. 2A,C, eIPSPs evoked by these interneurons were recorded within but not outside their axonal projection area. And as expected from monosynaptic GABAergic transmission, they displayed short latency (0.58±0.39 ms, n = 683 events, Fig. 2H,I).

### eIPSPs Mediate Powerful and Time Locked Feedback Inhibition in CA3

Basket cells are implicated in feedback inhibition, meaning that pyramidal cells firing triggers their discharge, which in turn provides inhibition [17]. Accordingly, firing of single action potentials by an intracellularly recorded CA3 pyramidal cell could evoke eIPSPs (n = 69 cells among 411 tested, see Fig. 2 for a representative example of one of the 8 labelled and morphologically identified CA3 pyramidal cells, based on their spiny basal and apical dendritic morphology, including thorny excrescences in stratum lucidum, and axon arborization) with a variable amplitude (Fig. 2F,G), and latencies larger than those of interneurons (2.30±0.71 ms, n = 796 events from 5 experiments, Fig. 2H,I; significantly different from interneuron-triggered eIPSP latencies, p<0.01, 2 sample Student t-test) suggestive of disynaptic mechanism [21], [22]. We next investigated quantitatively the interplay between recurrent excitation and feedback inhibition during spontaneous activity in CA3.

**Figure 3:**
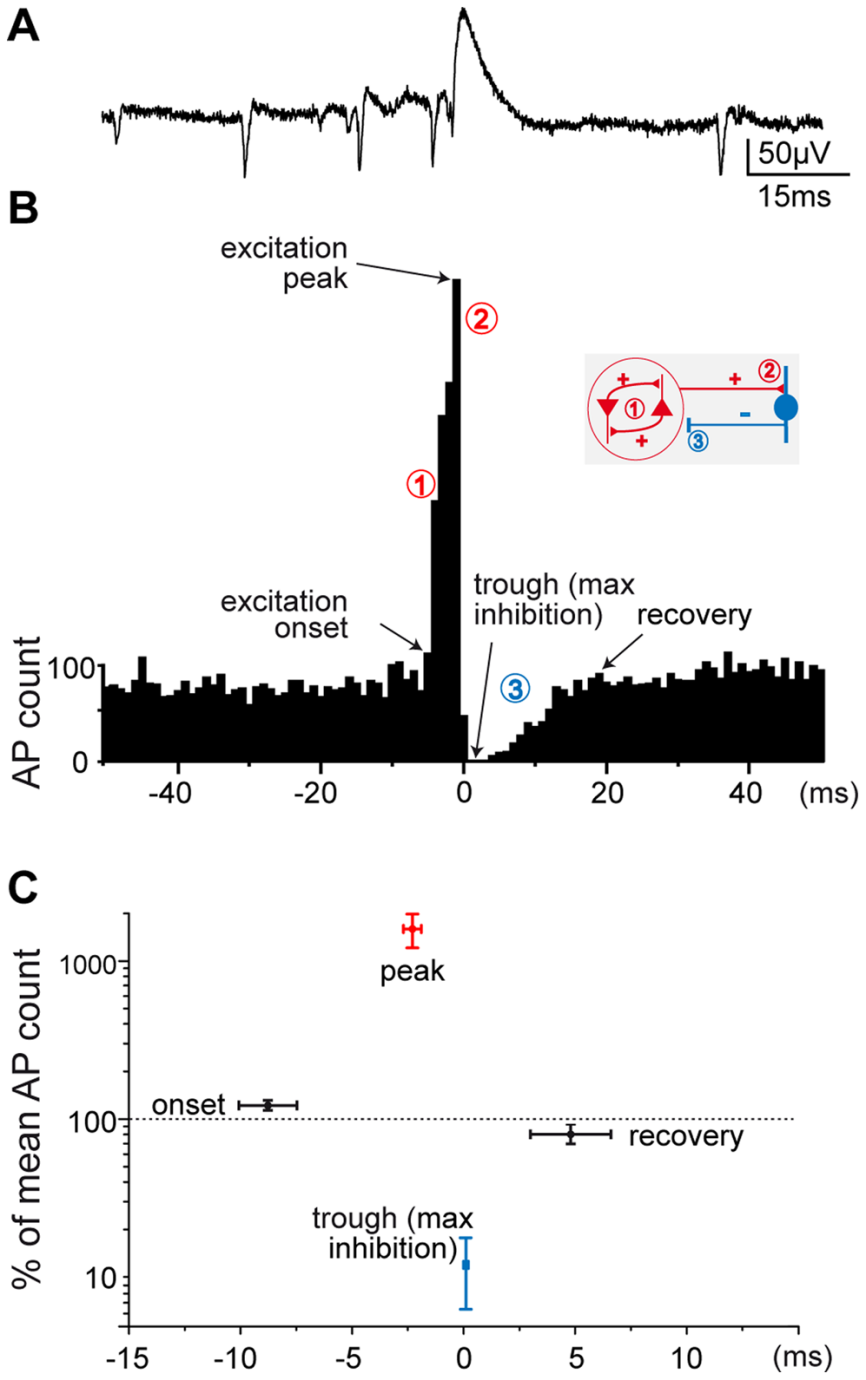
Recurrent excitation and inhibition ***in vitro*** **.** A. Example trace showing a spontaneous CA3 eIPSP and action potentials. B. Cross-correlogram (from a single representative experiment, in a guinea pig) between in vitro recorded multi-unit activity and eIPSP-peak time (used as reference). Time scales are similar in A and B, taken from the same recording, and eIPSP-peaks aligned for illustration. Time bin, 1 ms. Onset, first of pre-eIPSP time bins above mean +2SD; recovery, last of post-eIPSP time bins below mean - 2SD. C. Diagram illustrating firing rates (normalized and averaged across 9 slices from 3 guinea pigs, 3 mice and 3 rats, mean ± SEM, base line level = 100%) at onset, peak, trough, and recovery time points, as indicated in B. Note increased multi-unit discharge in the 10 ms prior to eIPSP, and the strong inhibition (**trough, max inhibition**) that follows. Inset, schematic illustration of the recurrent excitatory/inhibitory loop: (1) recruitment of interconnected pyramidal cells initiate collective discharge through recurrent excitation, (2) integration of excitation on a 10–15 ms time scale activates discharge of local interneurons, resulting (3) in perisomatic eIPSP and inhibition of pyramidal population activity.

To reveal the inhibition mediated by these interneurons during spontaneous activity, we examined cross-correlograms distributing multi-unit activity against perisomatic eIPSPs (Fig. 3). The post-eIPSP trough observed in the multi-unit distribution revealed a striking inhibitory effect on the local population discharge, with a brief but powerful inhibition following a time course similar to that of eIPSPs (Fig. 3, n = 9 animals), as previously reported for monosynaptic inhibitory connections in the neocortex [25]. Examination of population firing in the period immediately preceding eIPSPs reveals a peak in the crosscorrelogram (Fig. 3B), together with an increased spike count in the 10 ms preceding eIPSPs (pre-eIPSP firing above mean firing rate +2SD, 8.8±1.3 ms, n = 9 animals, mean ± SEM, Fig. 3C).

### Recruitment of Neuronal Firing and Inhibition in the CA3 Circuit

**Figure 4:**
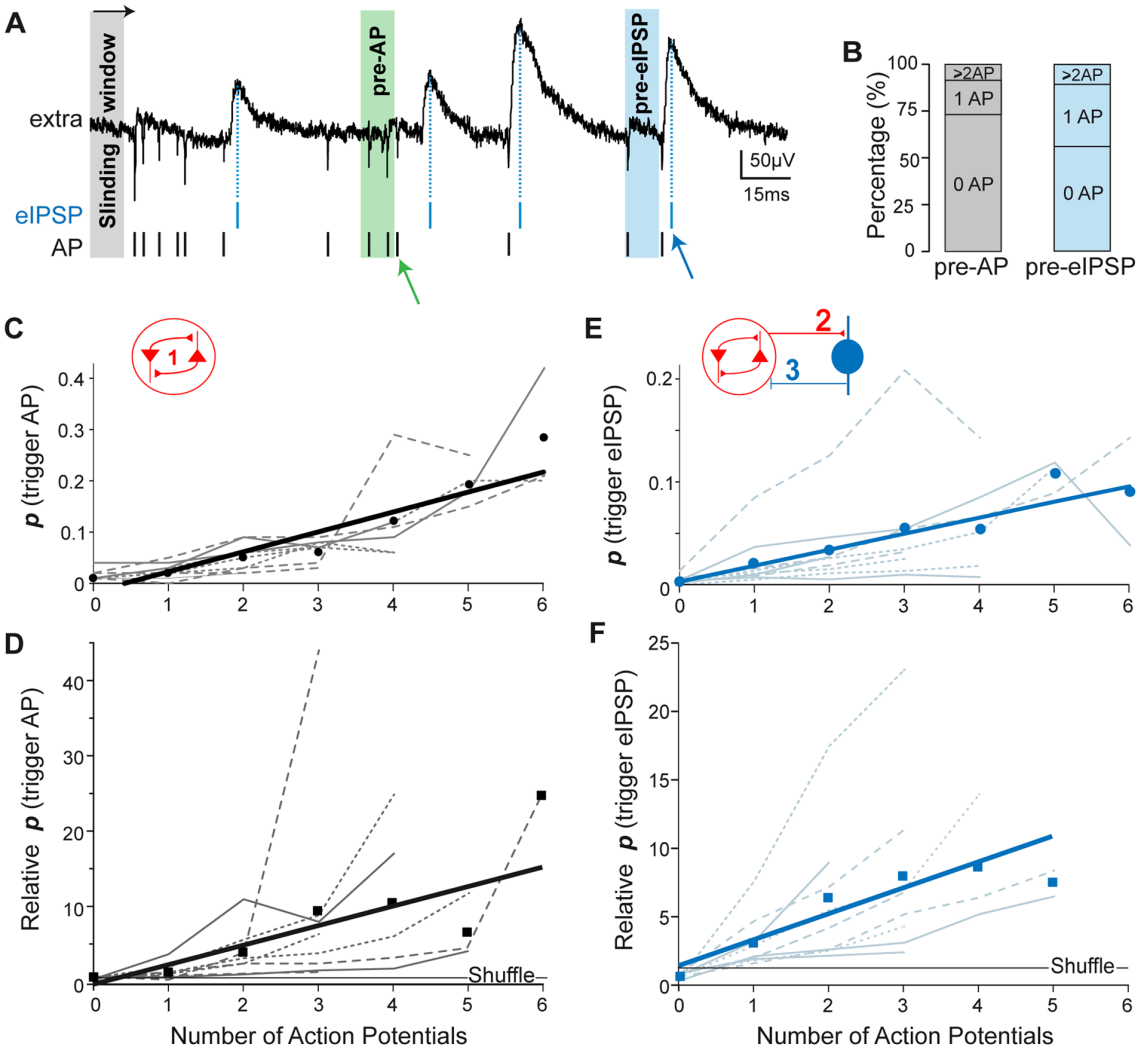
Recruitment of neuronal firing and inhibition in the CA3 circuit. A. The number of action potentials (AP, vertical bars and green arrow) within a 10 ms sliding window (time step 1 ms) was used to quantify the probability (see methods) for spike discharge to trigger action potentials (Pre-AP, light grey) or eIPSPs (Pre-eIPSP, light blue). Vertical blue line and arrow, eIPSP peak. B. Vertical bars show the probability (averaged from 9 animals) that an AP (left, grey) or eIPSP (right, blue) was preceded by a given number of spikes (within 10 ms). Note that only few (<10%) events (AP or eIPSPs) were preceded by more than 1 spike. C–F. Graphs showing the probability (absolute and relative to chance, see methods) that a given number of spikes (X axis) triggered a spike (**C, D**) or an eIPSP (**E, F**). Broken lines, individual animals (dotted lines, mice; dashed lines, guinea pigs rats; plain lines, rats); dots/squares, average values; thick lines, linear fit (see Table 1 for corresponding R^2^ and p values). Note the increasing probability of recruitment relative to chance (**D, F**), suggestive of neuronal cooperation in the circuit, and the modest absolute probabilities (<12% for eIPSP recruitment), suggestive of globally low levels of recruitment in the circuit.

Because we observed in the crosscorrelogram between MUA and eIPSPs that firing was increased during the 10 ms preceding eIPSPs, we hypothesized that 10 ms was a relevant time window to study the interactions between MUA and eIPSP occurrence. We therefore investigated in more detail the network firing dynamics on this time scale. In order to assess the potential role of recurrent excitation in hippocampal activity, we reasoned that if interconnected excitatory neurons entrain each other into collective discharge, the probability of recruiting additional spikes should increase with the number of discharging cells, a mechanism presumed to underlie seizure generation if not interrupted by inhibition. Estimating the relative probability of a given number of spikes to recruit additional spikes (see methods, Fig. 4), we indeed observed an increasing propensity to spike recruitment together with firing rate (for a 10 ms duration window, linear fit: R^2^ = 0.7, p<0.01, pooled data, n = 9 animals; for individual animals: R^2^ = 0.73±0.06, n = 8 animals, mean±SEM, Fig. 4D and Table 1), suggesting cooperativity between firing in this network. Nevertheless, the absolute probability for a group of spikes to recruit additional spikes remained relatively low (Fig. 4C), suggesting that the circuit remains under strong inhibitory control.

**Table 1:** Individual and pooled statistics for spike and eIPSP recruitment.

speciesanimal			mice			guinea pigs			rats			pooled
			1	2	3	1	2	3	1	2	3	n = 9
*p*(AP triggering AP)	Absolute	r^2^	0.8	0.7	0.9	0.7	0.4	0.9	0.6	0.8	0.5	**0.9**
		F	16	9	74	11	3	77	10	17	3	341
		*p*	*0.029*	*0.057*	*0.000*	*0.031*	*0.225*	*0.000*	*0.024*	*0.025*	*0.333*	*0.000*
			*		***	*		***	*	*		***
	Relative	r^2^	0.7	0.8	0.8	0.5	0.9	0.9	0.4	0.8	–	**0.7**
		F	12	11	26	4	28	57	5	16	–	113
		*p*	*0.038*	*0.081*	*0.007*	*0.189*	*0.034*	*0.001*	*0.082*	*0.026*	*–*	*0.000*
			*		**		*	***		*		***
*p*(AP triggering eIPSP)	Absolute	r^2^	1.0	0.9	0.8	0.6	1.0	0.9	0.3	0.3	0.9	**0.9**
		F	185	54	19	7	63	62	3	3	20	417
		*p*	*0.001*	*0.018*	*0.012*	*0.076*	*0.015*	*0.001*	*0.125*	*0.199*	*0.139*	*0.000*
			***	*	**		***	***				***
	Relative	r^2^	1.0	1.0	0.9	1.0	1.0	1.0	0.9	0.7	0.9	**0.9**
		F	197	41	34	234	80	192	92	6	19	231
		*p*	*0.005*	*0.023*	*0.010*	*0.004*	*0.012*	*0.000*	*0.001*	*0.242*	*0.144*	*0.000*
			**	*	*	**	*	***	***			***

Linear regression statistics parameters (**r^2^**, **F**, **p**; **Absolute** and **Relative** to shuffle: see methods) for the recruitment of action potentials (**p(AP triggering AP)**) and eIPSPs (**p(AP triggering eIPSP)**), for individual animals (**#1–3** in each species), and **pooled data**, as presented in Fig. 4C–F.

We next evaluated eIPSP recruitment in the network as the relationship between global instantaneous firing rate and occurrence of eIPSP. We found that the probability of triggering an eIPSP increased with firing rate (defined as the number of spikes within 10 ms, Fig. 4E–F, Table 1). Although very few eIPSPs were preceded by more than 2 spikes (<2%, Fig. 4B), this probability sharply increased relative to chance, from a factor of 3 for 1 spike to a factor of 8 for 4 spikes within 10 ms (Fig. 4F). However, the absolute probabilities remained very modest (<15%, Fig. 4E), suggesting that most CA3 circuit firing terminates for other causes than eIPSP-mediated, recurrent perisomatic inhibition.

### eIPSPs are Rare Events in CA1

**Figure 5:**
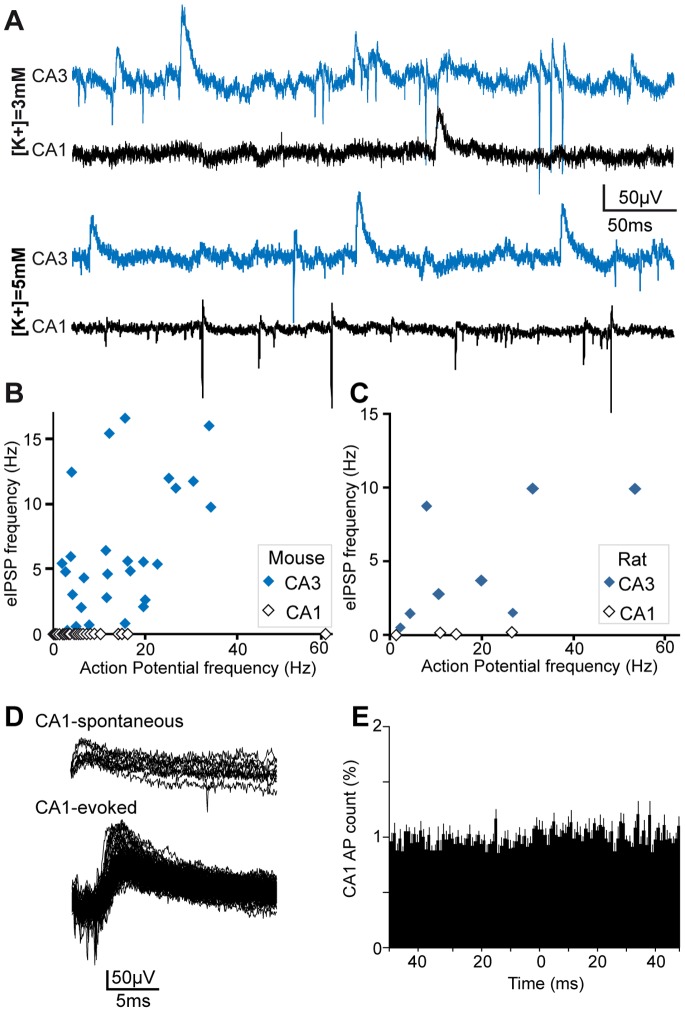
Spontaneous eIPSPs are rare events in CA1. A. Simultaneous recording of spontaneous activity in the CA3 and CA1 regions of a mouse in control (top traces, K^+^3 mM) and high K^+^ (lower traces, K^+^5 mM). The upper CA1 trace was specifically selected to illustrate a CA1 eIPSP, which were very rare events in CA1, even in presence of high-K^+^ induced intense multi-unit activity (lower CA1 trace). B–C. Relationship between multi-unit activity and eIPSP frequency (B, mice, n = 6; C, rats, n = 3), measured in CA3 (blue diamonds) and CA1 (white diamonds) simultaneously, in presence of various extracellular K^+^ concentrations (range 3–8 mM). Note that eIPSP frequency in CA1 remains low whatever the frequency of multi-unit activity. D. Superimposed traces of rat CA1 eIPSPs that either occured spontaneously (CA1-spontaneous, upper traces) or were evoked by minimal extracellular electrical stimulation close to the CA1 pyramidal layer (CA1-evoked, lower traces). E. Crosscorrelogram between CA1-MUA and CA3-eIPSPs (rats, n = 3).

In order to extend our investigation to a different microcircuit, we examined the spontaneous expression of eIPSPs in CA1. Interestingly, although evoked eIPSPs have been described in both CA1 [21] and CA3 [22] hippocampal subfields, we have observed that during spontaneous activity, recorded simultaneously from CA1 and CA3 pyramidal layers, eIPSPs that are common in CA3 were rare events in CA1 (for mice, 0.002±0.002 Hz in CA1 vs 3.6±2 Hz in CA3, n = 6, p<0.01, paired student t-test; for rats, 0.13±0.1 Hz in CA1 vs 4.8±4 Hz in CA3, n = 4, p<0.05, paired student t-test), even in the presence of increased extracellular K^+^ concentration to increase spontaneous activity (Fig. 5).

Cross-analysis of CA3 eIPSPs and CA1 MUA did not show a significant influence of eIPSP-related CA3 activity on CA1 neuronal firing (Fig. 5E). Due to the infrequent occurrence of eIPSPs in CA1, we did not investigate the influence of CA1 eIPSPs on local CA1 MUA.

Because previous studies suggest a more hyperpolarized resting membrane potential in CA1 compared to CA3 pyramidal neurons [26], [27], the measured low frequency of spontaneous eIPSPs in CA1 might be due to smaller Cl^-^ driving force in CA1 and hence eIPSP amplitude falling below our detection threshold. Firstly, even in the presence of high (5–8 mM) extracellular K+, a condition of presumed depolarized membrane potential as suggested by the highly increased firing rate (from 2.7±4 Hz in control to 11.6±25 Hz for 6 mM K+, n = 6), CA1 eIPSP frequency remained much lower than in CA3 (Fig. 5A–C). Secondly, eIPSPs of amplitudes clearly above signal to noise ratio could be evoked in CA1 by minimal electrical stimulation close to stratum pyramidale, in the presence of the AMPA-receptor blocker NBQX (Fig. 5D). Therefore, eIPSPs corresponding to GABA-A R mediated postsynaptic pyramidal population responses to presynaptic basket cell firing can be recorded during spontaneous activity in the CA1 and CA3 pyramidal layers of hippocampal slices. However, our results raise the interesting hypothesis that their participation to the regulation of spontaneous activity might be much more prominent in CA3, a circuit characterized by the presence of excitatory recurrent collaterals and in which tight inhibitory control is particularly crucial. Further investigation of basket cell firing recruitment during spontaneous activity, including in vivo, will be necessary to investigate this potential difference between CA1 and CA3 circuits in more detail.

## Discussion

Our results confirm recently published data that currents mediated by the activation of GABA-A receptors on the pyramidal-cell target-population of individual perisomatically projecting interneurons can be recorded as positive LFP in CA3 and CA1 pyramid layer [21], [22]. Nevertheless, while eIPSPs were spontaneously expressed at high rate in the CA3 region, they participated very little to the spontaneous activity in CA1. Further studies will be necessary to understand these regional differences, which potentially include lower excitability of CA1 interneurons, less efficient pyramid to interneuron excitatory synapse, or lower pyramidal cell to interneuron connectivity in CA1.

### Potential Relevance for Neuronal Coding and Consolidation

Analysing the temporal relationships between spiking activity and eIPSPs as an index of CA3 network dynamics may provide a better understanding of neuronal coding and information storage into neuronal networks. According to the cell assembly hypothesis, the neurons representing the various features of a cognitive item are assumed to fire together and reinforce their interconnections, so that subsequent activation of a subset of this group can recruit the remaining participants and reconstitute the original cell assembly[6], [8], [28]–[40]. This property is also the theoretical basis for pattern completion, a function attributed to the CA3 network [30], [31], [41]. In this respect, neurons belonging to the same cell assembly need to be interconnected and to fire together within a time window that matches the constraints of Hebbian synaptic plasticity. It is therefore crucial to know whether CA3 neurons do recruit each other during spontaneous activity, in presence of functional GABAergic inhibition.

Previous work [42] pointed out that during sharp-wave ripples (SPW-ripples), an intermittent pattern associated with information replay and consolidation which occurs mainly during specific behavioural states such as immobility and slow wave sleep, CA3 and CA1 hippocampal regions transiently showed higher levels of synchrony (proportion of neurons firing together within 100 ms time windows) than expected from a random occurrence of spikes, given the estimated individual neurons’ firing rates. Although this can be interpreted as evidence for neuronal cooperation, it can hardly be attributed unequivocally to CA3 recurrent collaterals because hippocampal inputs from the entorhinal cortex, which are also active during SPW-ripples, might also be involved in the increased synchrony observed in CA3 and CA1 regions. CA3 is the only hippocampal region presenting excitatory recurrent connections [12], and in presence of GABAergic blockers, CA3 pyramidal cells readily recruit each other into hyper-synchronized epileptiform discharges [9], [11]. However, it was also observed that in the absence of GABAergic blockers, firing of CA3 pyramidal cells was rarely preceded by an excitatory post-synaptic-potential (EPSP), as opposed to the firing of interneurons that was almost systematically preceded by an EPSP, suggesting tight inhibitory control of network excitability by feedback inhibition and poor recruitment of pyramidal neurons through recurrent connections in the presence of ongoing inhibition [17], [43]. Therefore, it remained unclear whether interconnected CA3 neurons do recruit each other during ongoing activity.

### Neuronal Recruitment into Collective Discharges

While we presently confirm the previous report that pyramidal cells can recruit feedback inhibition [17], our results showing increasing relative probability of spike recruitment with instantaneous firing rate are likely to reflect involvement of CA3 excitatory recurrent collaterals, because there is virtually no external input activity to CA3 in hippocampal slices (perforant path is disrupted and Dentate granule cells are quiescent in slices). Previous studies [44]–[46] suggested that pyramidal cells can recruit each other through recurrent collaterals in CA3 within about 12 ms, and can recruit recurrent inhibition within about 3.5 ms. This is in a time range compatible with the 10 ms time window we report here. Nevertheless, our results using extracellular recordings of spontaneous activity are mostly based on correlative analysis of neuronal firing, and we can not exclude alternative possibilities such as synchronous disinhibition of pyramidal cells and perisomatically projecting interneurons.

Multi-unit activity recordings do not allow to separate the firing from individual neurons and may include bursts of spikes fired by the same neuron. In our study, spike recruitment within the CA3 circuit therefore refers to the global amount of activity within this network, including burst discharge by individual neurons.

Interestingly, we also observed that CA3 neuronal cooperation operates on a short time scale (10 ms), relevant for Hebbian synaptic plasticity, and in absence of SPW-ripples, which is actually consistent with the current view that SPW-ripples would consolidate cell assemblies expressed during other behaviours, and with the previous report that replay of awake activity is also expressed in non SPW-ripple epochs, such as REM sleep [47] .

### Inhibitory Control of Collective Discharges

It was verified both theoretically and experimentally that as a counterpart to recurrent excitatory connections, Hebbian (or auto-associative) neuronal networks are prone to hyper-synchrony and pathological seizures [9], [11], [13], [30]. Therefore in such networks, the expression of cell assemblies requires tight inhibitory control to set limits to the number of simultaneously discharging neurons. Our results suggest that although there is a cooperativity between cellular firing to recruit feedback inhibition (increasing eIPSP recruitment probability together with firing rate, relative to chance), eIPSPs remain unreliably evoked by neuronal discharge, presuming a limited role of feedback inhibition from perisomatic eIPSPs in the inhibitory control of firing runaway. Because time locked inhibition by eIPSPs is striking but eIPSPs are rather poorly recruited by network activity, our expectation is that eIPSPs-related perisomatic inhibition should be more efficient in shaping the timing of spike flow than in limiting excitatory runaway recruitment and preventing the generation of hyper-synchronized discharges. Previous report that transgenic mice with specifically disrupted glutamatergic inputs to parvalbumin-positive interneurons displayed hippocampo-dependent spatial memory impairment but no epileptic phenotype are in support of this interpretation [48], [49].

### Conclusion

In order to understand the functioning of a neuronal network, it is essential to have a precise knowledge of how neurons interact, meaning the nature and dynamics of synaptic interactions and integration. In this study, we provide quantified parameters of neuronal interactions in the hippocampal network, which may prove useful for the identification of network correlates of the numerous neuronal pathologies affecting hippocampo-dependent cognitive abilities, such as epilepsy, Alzheimer and Huntington diseases, as well as senility.

## Materials and Methods

All animal experiments were performed following INSERM guidelines and the official French veterinary regulation concerning animal experimentation (decret 87–848, 10/19/1987).

### *In vitro* Recordings

Hippocampal slices from guinea pigs (n = 10, age 4–6 weeks), mice (n = 10, age P20–25) and rats (n = 52, age P21–30) were prepared as described elsewhere [17]. Because similar results were obtained with mice, guinea pigs and rats (Table 2), these were pooled together unless otherwise stated. In brief, animals were anesthetized either with a cocktail of ketamine-xylazine (respectively 100 mg/kg and 20 mg/kg, I.P. injection) or with urethane (1.5 g/kg, I.P. injection), and perfused intracardially with ice cold modified ACSF of the following composition (mM): sucrose (248), KCl (4), CaCl_2_ (1), MgCl_2_ (5), NaHCO_3_ (26), glucose (10). The brain was then removed and cut into 500 µm thick slices, perpendicular to the longitudinal hippocampal axis (transverse slices) using a vibratome. The slices were positioned on a nylon mesh in a beaker or in the recording chamber and kept at the interface between ACSF (composition NaCl (124), KCl (3), CaCl_2_ (3), MgCl_2_ (2), NaH_2_PO_4_ (1), NaHCO_3_ (26), glucose (10)) and humidified gas (95% O_2_, 5% CO_2_) at 36°C. Recordings started after a 1- to 2-hour recovery period.

**Table 2:** Species comparison of AP and eIPSP frequencies.

	mice vs g.pigs		rats vs g.pigs		mice vs rats	
	t	*p*	t	*p*	t	*p*
CA3 AP frequency (Hz)	−1.77	*0.15*	−0.13	*0.90*	0.77	*0.48*
CA3 eIPSP frequency (Hz)	−1.18	*0.30*	0.27	*0.80*	0.85	*0.44*
Cross-corr AP count onset Ex (%)	0.03	*0.98*	0.40	*0.71*	−0.60	*0.58*
Cross-corr AP count peak Ex (%)	0.46	*0.67*	1.02	*0.37*	−0.87	*0.43*
Cross-corr AP count max Ib (%)	0.50	*0.64*	−1.21	*0.29*	−1.21	*0.29*
Cross-corr AP count recovery (%)	0.76	*0.49*	0.61	*0.58*	0.04	*0.97*
Cross-corr time onset Ex (ms)	−1.08	*0.34*	0.99	*0.38*	0.20	*0.85*
Cross-corr time peak Ex (ms)	−5.00	0.01**	1.41	*0.23*	0.38	*0.72*
Cross-corr time max Ib (ms)	−1.73	*0.16*	1.51	*0.21*	−0.50	*0.64*
Cross-corr time recovery (ms)	−1.91	*0.13*	2.43	*0.07*	−1.41	*0.23*

Two sample Student’s t-test (t, p) comparing various parameters in mice (n = 3), guinea pigs (n = 3) and rats (n = 3).

CA3 AP frequency: mean firing rate; CA3 eIPSP frequency: mean frequency of eIPSP occurence.

cross-corr AP count: % of mean AP count in the considered bin (onset Ex, peak Ex, max Ib, recovery: cf Methods and Fig. 3).

cross-corr time: time of occurence of the considered bin (onset Ex, peak Ex, max Ib, recovery: cf Methods and Fig. 3).

onset Ex: excitation onset bin.

peak Ex: excitation peak bin.

max Ib: maximal inhibition bin.

recovery: recovery bin.

Note that besides the time of occurence of the excitation peak in mice compared to guinea pigs, none of the tested values were significantly different between species.

Extracellular recordings of spontaneous multi-unit activity and local field potentials were performed using 1 to 4 single isolated wires (tungsten coated with heavy butyral bond, diameter 20–50 µm) mounted on individual micromanipulators (Narishige, Japan) and connected to extracellular amplifiers (Knight/Neuralynx L8). The signal was digitized using a Digidata A/D converter (Axon Instruments) and stored on a PC for offline analysis. In some experiments, a bipolar stimulation electrode made of 2 intermingled tunsgten wires (40 µm diameter, formward coating) was positioned close to the CA1 pyramidal layer. Electrical stimulations (15 to 80 µA, 10 to 30 µs duration) were delivered at a rate of 1 Hz, in the presence of the AMPA-receptor blocker NBQX to prevent disynaptic recruitment of inhibitory interneurons. Various stimulation intensities were tested and recording was done with the lowest intensity eliciting detectable eIPSP responses. Intracellular recordings were performed using borosilicate capillaries pulled on a Sutter P87 microelectrode puller and filled with 3 M K-Glu or 2 M K-AC. In some experiments, dextran rhodamine (5%), biocytin (0.3 to 1.5%) or neurobiotin (1.5%) was added to the internal solution. Patch clamp whole cell recordings were performed using 10–15 MΩ pipettes filled with internal solution of the following composition (mM): K-MeSO_4_ (135), Mg-ATP (2), EGTA (1), HEPES (10), MgCl_2_ (2), CaCl_2_ (0.1).

### Anatomical Analysis

To analyse the morphology of the cells that trigger eIPSPs, the slices with biocytin- or neurobiotin-filled neurons were fixed in 0.1 M phosphate buffer containing 2% paraformaldehyde and 0.1% picric acid for 2 days at 4°C and resectioned at 60 µm thickness. Some of the sections were analyzed both by immunohistochemistry and by subsequent diaminobenzidine (DAB) reaction, while in the rest of the experiments the identity of the recorded cells were defined by DAB reaction alone.

For immunohistochemistry, sections were incubated with primary antibodies raised against parvalbumin (Swant PV28; 1∶1000, polyclonal rabbit) and somatostatin (Millipore Bioscience Research Reagents; MAB354; 1∶500, monoclonal rat, YC7) overnight in 0.3% Triton X-100 and 2% normal goat serum containing TBS buffer at 4°C. Immunoreactions were revealed using goat anti-rabbit IgG Alexa 488- or anti-rat IgG Alexa 594-conjugated antibodies (Invitrogen), and neurobiotin or biocytin staining was revealed using Alexa 350-conjugated streptavidin. Sections were mounted with Vectashield media.

In order to reveal the fine details of the morphology of the cells we used a conventional DAB staining method which either followed the immunohistochemical reactions or was carried out immediately after resectioning. After washing the sections in phosphate buffer, endogenous peroxidase activity was blocked with 1% H_2_O_2_. Sections were then washed again and incubated overnight with ABC (avidin-biotin complex) reagent (Vectastain ABC Standard Elite kit, Vector Laboratories) in 0.1% Triton X-100 containing buffer, at room temperature. The reaction was developed with DAB and NiCl_2_ for 8–10 min and stopped with H_2_O_2_ solution. Sections were dehydrated (50, 70, 90, and 95%, 2× absolute ethanol and 2× Sigma HistoChoice solutions for 10 minutes each) on slides and mounted using DPX mounting media. For drawing of the cells we used conventional camera lucida with 100× objective (Leica DM2500 microscope).

### Data Analysis

Spontaneous eIPSPs were detected with Detectivent software (developed under Labview and kindly provided by N. Ankri). After low pass filtering with Bessel or Butterworth filters (upper threshold between 250 and 400 Hz), an up-only transform was applied and signal rises above an adjustable threshold were detected [22], [23] and considered as putative eIPSPs. Event amplitude was measured from the original signal, and event timing defined as the event peak. Under visual inspection, thresholds were set for automatic eIPSP detection on amplitude (2 to 4SD above noise level), time to peak (>1 to 1.5 ms) and decay time constant (>0.1 ms).

Multi-unit activity was detected using nd-manager free-software (by J. Csicsvari, K.D. Harris, L. Hazan & M. Zugaro). After high pass filtering (>800 Hz) and signal integration, all events above 1.5 SD of signal fluctuations were detected as putative action potentials (AP). Semi-automatic clustering based on principal component analysis of wave shapes (K.D. Harris) was used to separate multi-unit activity from recording artefacts.

Crosscorrelograms compare the distribution in time of multi-unit activity and eIPSPs detected from the same electrode. The number of spikes was counted in time-bins of 1 ms in the interval −50 to +50 ms around eIPSP times (reference, time 0), and normalized by dividing the values in each bin by the average number of spikes per bin. Baseline fluctuations (mean and SD) were calculated from the time interval −50 to −15 ms. Excitation-onset corresponds to the first time-bin in which the number of spikes is above mean-baseline +2SD (ie. 95% confidence interval that firing in this time bin is higher than baseline). Excitation-peak corresponds to the time-bin after excitation-onset that contains the maximum number of spikes. Maximal inhibition (trough) corresponds to the post-peak time-bin containing the minimal number of spikes. The recovery point corresponds to the first time-bin after maximal inhibition in which the number of spikes reaches baseline - 2SD.

Spike-train quantification was performed by counting the number of action potentials within a sliding window (duration 10 ms, time step 1 ms, Fig. 4A), an equivalent to instantaneous firing rate measured at a time interval of 1 ms and smoothed on a 10 ms time scale. Overall, the number of action potentials within 10 ms ranged between 0 and 6. We investigated the probability that a given “instantaneous firing rate” (that is n AP within 10 ms) triggers (or immediately preceded) an event such as an action potential or an eIPSP.

(a) action potential recruitment: The action potentials fired within a given 10 ms sliding window were considered as having potentially recruited an additional spike if an AP occurred within the 1 ms that followed the end of the 10 ms sliding window. The absolute probability of n AP to recruit an additional spike was calculated as the ratio between “the number of windows containing n AP and followed by an additional AP within 1 ms” and “the total number of windows containing n AP”. The corresponding formula is:
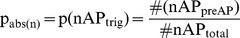
with Pabs(n) = p(nAPtrig) = absolute probability that n AP triggered additional firing

#(nAPpre-AP) = number of windows containing n AP and preceding a spike in the coming 1 ms after the end of the time window.

#(nAPtotal) = total number of time windows containing n AP.

We observed that this probability increased with firing rate (ie. the more spikes within 10 ms, the more chance that an addition spike will follow), suggesting positive interaction between firing rate and spike recruitment (ie blazing up: the more intense the firing, the more chance to recruit additional firing).

(b) eIPSP recruitment: The action potentials fired within the 10 ms sliding window that corresponds to the time interval [−12 ms, −2 ms] preceding an eIPSP were considered as having potentially recruited an eIPSP. The absolute probability of nAP to recruit an eIPSP was calculated as the ratio between “the number of windows containing n spikes and corresponding to the time interval [−12 ms, −2 ms] preceding an eIPSP” and “the total number of windows containing n spikes”. The corresponding formula is:
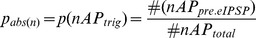
where Pabs(n) = p(nAPtrig) = absolute probability that n AP triggered an eIPSP event

#(nAPpre-eIPSP) = number of 10 ms sliding windows containing n AP and corresponding to the time interval [−12 ms, −2 ms[preceding an eIPSP.

#(nAPtotal) = total number of 10 ms sliding windows containing n AP.

The reason why we considered the time interval [−12, −2[ms preceding an eIPSP, rather than the interval [−11, −1[ms as used for AP recruitment, is that it allows to exclude from the spike count of the succesfully triggering 10 ms sliding windows the spikes fired at monosynaptic latency from eIPSPs (2 ms, cf Fig. 2). We chose this option to favor the quantification of pyramidal cell participation (which is di-synaptic, cf Fig. 2) to eIPSP recruitment.

(c) relative probabilities: To assess the statistical significance and eliminate potential biases in the quantification of the absolute experimental probabilities described in (a) and (b), the Inter-Spike Intervals of the original spike train were randomly shuffled (circular permutation) to generate 100 surrogate spike trains. The analysis presented above were conducted for each of these surrogate spike trains, keeping the experimental eIPSP dates. The resulting probabilities are averaged for direct comparison with experimental observations, leading to relative probabilities (expressed as the ratio between experimental and shuffled calculated probabilities). The corresponding formulas are:
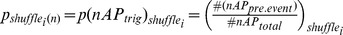
where Pshuffle-i(n) = p(nAPtrig)shuffle-i = absolute probability that in the surrogate spike train i (i from 1 to 100 since we generated 100 surrogate spike trains) nAP triggered an event (either spike for spike recruitment quantification or eIPSP for eIPSP recruitment quantification)



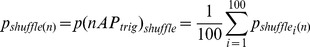
where Pshuffle(n) = p(nAPtrig)shuffle = averaged absolute probabilities, calculated from the 100 surrogate spike trains, that nAP triggered an event



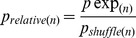
where Prelative(n) = relative probability (experimental compared to shuffle) that nAP triggered an event (either AP or eIPSP)

Pexp(n) = absolute probability calculated from the experimental spike train that nAP triggered an event.

Pshuffle(n) = absolute probability calculated (and averaged) from the 100 surrogate spike trains that nAP triggered an event.

Comparisons were limited to spike rates (n spikes within 10 ms) for which the number of occurrences (sample size) was high enough to allow for reliable statistical analysis, as determined by the binofit Matlab function (at confidence level >95%). Sliding windows of various durations (between 6 and 30 ms) were tested. Across individual animals, the overall strongest recruitment occurred on a time scale of 10 ms for eIPSPs and action potentials (data not shown).

In most experiments, we found statistically significant (p<0.05) linear fit between instantaneous firing rate and the relative probability of triggering an event (eIPSP or action potential). Linear regression parameters were estimated for both pooled data and individual animals (Table 1) using Origin Software (OriginLab Co.).

Unless stated otherwise, all statistics are given as mean ± SD.
